# Prolonged Nocturnal Sleep Duration as a Risk Factor for Cognitive Decline in Elderly Chinese Adults: A Post Hoc Cohort Analysis

**DOI:** 10.1002/alz70860_101970

**Published:** 2025-12-23

**Authors:** Hetong Ma, Xin Gao, Haoran Chen, Zhaoxue Yin, Xiang Si, Yibing Yang, Zhen Guo, Jiao Li, Jianqiang Lai

**Affiliations:** ^1^ Institute of Medical Information/Library, Chinese Academy of Medical Sciences/Peking Union Medical College, Beijing, Beijing, China; ^2^ Chinese Center for Disease Control and Prevention, Beijing, Beijing, China

## Abstract

**Background:**

Emerging evidence highlights a bidirectional relationship between sleep and cognitive health. Cross‐sectional studies have identified associations between abnormal sleep patterns (e.g., short/long duration, fragmented sleep) and neurodegenerative processes, though findings remain inconclusive. This study addresses this gap by examining nocturnal sleep phenotypes and their impact on cognitive trajectories in a nationally representative cohort of Chinese older adults. Education‐adjusted cognitive thresholds were implemented to account for China's significant educational disparities among elderly populations.

**Method:**

This analysis utilized data from a prospective cohort study spanning six Chinese provinces, encompassing 3,428 community‐dwelling adults aged ≥65 years (baseline: 2019; follow‐up: 2022). Cognitive assessment employed the Mini‐Mental State Examination (MMSE), with impairment defined using education‐stratified cutoffs: ≤19 for illiterates, ≤22 for primary education, and ≤26 for secondary education or higher. Sleep parameters were derived from validated questionnaires assessing. Nocturnal sleep phenotype was categorized as short (<7 hr), normative (7‐9 hr; reference), or prolonged (≥9 hr). Sleep satisfaction and sleep duration were also collected. Multivariable logistic regression models were employed for cross‐sectional analysis.

**Result:**

In the 2019 survey, 26.7% (914) of older adults exhibited cognitive impairment. Sleep duration data revealed that 1,337 participants slept less than 7 hours per night, 1,605 slept 7‐9 hours (the recommended range), and 486 slept 9 hours or more. The cross‐sectional analysis indicated that normative sleepers (7‐9 hr/night) demonstrated significantly decreased odds of cognitive impairment compared to prolonged sleepers (≥9 hr/night).

**Conclusion:**

This study identifies prolonged nocturnal sleep (≥9 hr) as an independent risk marker for cognitive decline in Chinese elderly. Clinicians should consider sleep pattern monitoring as part of dementia risk stratification strategies. Further investigation is warranted to determine whether sleep duration optimization could modify cognitive trajectories in at‐risk populations.

